# Supplementary material for: Effectiveness of Dengue Awareness Calendar on Indigenous Population: Impact on Knowledge, Belief and Practice
Source: Healthcare (Basel). 2023 Feb 21;11(5):637. doi: 10.3390/healthcare11050637 (PMC10000413; doi:10.3390/healthcare11050637)
Supplement: Supplementary file 1 [file healthcare-11-00637-s001.zip › Figure S2 The percentage of correct responses of dengue knowledge items pre- and post-intervention .pdf]

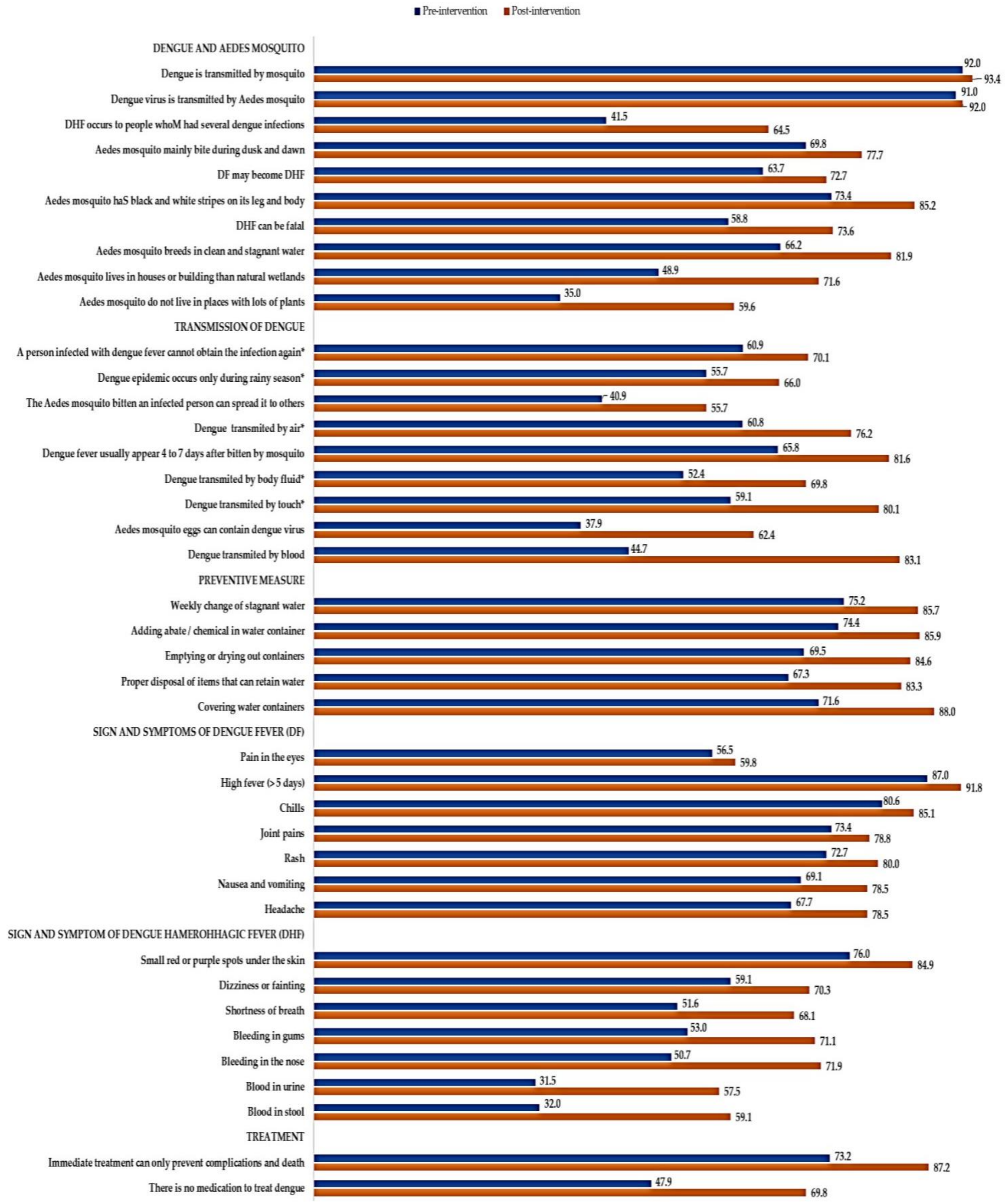

Figure S2: The percentage of correct responses of dengue knowledge items pre- and post-intervention (N= 609)
