# Supplementary material for: Effectiveness of Dengue Awareness Calendar on Indigenous Population: Impact on Knowledge, Belief and Practice
Source: Healthcare (Basel). 2023 Feb 21;11(5):637. doi: 10.3390/healthcare11050637 (PMC10000413; doi:10.3390/healthcare11050637)
Supplement: Supplementary file 1 [file healthcare-11-00637-s001.zip › File S1 Survey Questionnaire.pdf]

KNOWLEDGE, ATTITUDE AND PRACTICES REGARDING DENGUE FEVER AMONG ORANG ASLI COMMUNITY IN MALAYSIA (PRE-INTERVENTION)

**Section A: Socio-Demographic**		
A. 1	Age	________________years old
A. 2	Gender	☐ Male<br>☐ Female
A. 3	Name of the tribe	___________________
A. 4	Highest educational level	☐ No formal education<br>☐ Primary school<br>☐ Secondary school<br>☐ Tertiary
A. 5	Occupation	☐ Professional and managerial<br>☐ Manual worker<br>☐ Housewife<br>☐ Student<br>☐ Retired<br>☐ Others;________________________
A. 6	Monthly average household income	RM________________/ month
**Section B: Dengue Experience**		
B. 1	Have you ever had dengue fever?	☐ Yes<br>☐ No
Section C: Mosquito Problem in Neighborhood Area		
C. 1	Does your house have a lot of plants or vegetation?	☐ None<br>☐ Low<br>☐ Moderate<br>☐ A lot
C. 2	How would you rate the mosquito problem in your neighborhood?	☐ None<br>☐ Low<br>☐ Moderate<br>☐ Severe
C. 3	Frequency of mosquito fogging in your neighborhood.	☐ None<br>☐ Rarely<br>☐ Occasionally<br>☐ Often

<table>
<tr><td colspan="5">Section D: Health Belief Model</td></tr>
<tr><td>D. 1</td><td>Do you think dengue is dangerous? Please state from scale 1(not dangerous) to 10 (very dangerous)</td><td colspan="3">0 1 2 3 4 5 6 7 8 9 10
NOT dangerous … VERY dangerous</td></tr>
<tr><td>D. 2</td><td>From scale 1 (very unlikely) to 10 (highly likely), how do you rate your likelihood of getting dengue (risk of getting dengue)?</td><td colspan="3">0 1 2 3 4 5 6 7 8 9 10
VERY unlikely … HIGH likely</td></tr>
<tr><td>D. 3</td><td>From scale 1 (no barriers) to 10 (high barrier), how do you rate the barriers you face to prevent dengue?</td><td colspan="3">0 1 2 3 4 5 6 7 8 9 10
NO barriers at all … HIGH barrier</td></tr>
<tr><td>D. 4</td><td>From scale (Definitiely do no take action) to 10 (Definitely do the action), how do you rate any cues to action – incidence that motivate someone to carry out the prevention practices like death of family member due to dengue, encouragement from NGO, neighborhood infection with dengue.</td><td colspan="3">0 1 2 3 4 5 6 7 8 9 10
DO NOT take action … DO the action</td></tr>
<tr><td>D. 5</td><td>From scale 1 (very not confident) to 10 (very confident), please rate your confidence level to carry out dengue prevention activities.</td><td colspan="3">0 1 2 3 4 5 6 7 8 9 10
No confident at all … VERY confident</td></tr>
<tr><td></td><td></td><td colspan="3"></td></tr>
<tr><td colspan="5">Section E: Knowledge Related to Dengue</td></tr>
<tr><td></td><td>Statements</td><td>Yes</td><td>No</td><td>Don’t Know</td></tr>
<tr><td colspan="5">General Knowledge about dengue and Aedes Mosquito</td></tr>
<tr><td>E. 1</td><td>Dengue is transmitted by mosquito</td><td></td><td></td><td></td></tr>
<tr><td>E. 2</td><td>The dengue virus is transmitted by Aedes Mosquito</td><td></td><td></td><td></td></tr>
<tr><td>E. 3</td><td>Dengue Fever may become <u>Dengue Hemorrhagic Fever</u></td><td></td><td></td><td></td></tr>
</table>

E. 4	Dengue hemorrhagic fever can be fatal			
E. 5	Dengue Hemorrhagic Fever usually occurs in people who have had several dengue infections			
E. 6	Aedes mosquitos have black and white stripes on its leg and body			
E. 7	Aedes mosquito breeds in clean and stagnant water			
E. 8	Aedes mosquito prefers to live in the house or building rather than in natural wetlands			
E. 9	Aedes mosquito prefers to live in places with a lot of plants			
E.10	Aedes mosquitoes mainly bite during dusk and dawn			
**Knowledge about the transmission of dengue**				
E.11	The Aedes mosquitoes biting an infected person can spread it to another person			
E.12	Dengue fever usually appear 4 to 7 days after someone has been bitten by mosquito			
E.13	Dengue disease can be transmitted from an infected person by<br>touching			
E.14	Dengue disease can be transmitted from an infected person by Air			
E.15	Dengue disease can be transmitted from an infected person by Body fluid (saliva, sweat, semen)			
E.16	Dengue disease can be transmitted from an infected person by Blood			
E.17	Aedes mosquitoes’ eggs can contain the dengue virus			
E.18	A person who has had dengue fever cannot get the infection again			
E.19	Dengue epidemic occurs only during the rainy season			
**Knowledge about prevention**				
E.20	Breeding of Aedes mosquitoes in the house can be prevented by<br>weekly change of stagnant water (pet bowls, flower pots or vases,etc.) in and around the house.			
E.21	Breeding of Aedes mosquitoes in the house can be prevented by<br>adding Abate/chemical in water containers			
E.22	Breeding of Aedes mosquitoes in the house can be prevented by covering water containers			
E.23	Breeding of Aedes mosquitoes in the house can be prevented by periodically emptying or drying out			

	containers (old tires, trash cans, coconut shell etc) that retain water in and around the house			
E.24	Breeding of Aedes mosquitoes in the house can be prevented by proper disposal of items that can retain water (old tires, trash cans, coconut shell etc) around the house			
**Signs and symptoms of dengue fever**				
E.25	The high fever for 5 to 6 days			
E.26	Chills			
E.27	Rash			
E.28	Pain in the eyes			
E.29	Joint pain			
E.30	Headache			
E.31	Nausea and vomiting			
**Signs and symptoms of Dengue Hemorrhagic Fever (DHF)**				
E.32	Small red or purple spots under the skin			
E.33	Bleeding in the nose			
E.34	Bleeding in the gums			
E.35	Blood in stool			
E.36	Blood in urine			
E.37	Shortness of breath			
E.38	Dizziness or fainting			
**Knowledge about treatment, curability and precaution measures for people infected with dengue**				
E.39	There is no medication for treating dengue			
E.40	Immediate treatment can only prevent complications and death			

**Section F: Practices Regarding Dengue Prevention**					
	**Statements**	**Not at all**	**Rarely**	**Sometimes**	**Often**
**Prevention of Mosquito Breeding**					
F. 1	Cover all water containers used for storing water in or outside the house				
F. 2	Change stored water i.e water in water tank, pails, bathtub, flower vases, money plant, plates that support plant pots, refrigerator drip tray, plastic container on the bottom of kitchen cabinet/table legs (for controlling ants), etc.				
F. 3	Put Abate or chemical in water storage containers				

F. 4	Examine for mosquito larvae in containers for storing water, eg. pails, flower vases, water in money plant, plates that support plant pots, refrigerator drip tray, plastic container on the bottom of kitchen cabinet/table legs, etc.				
F. 5	Clear out debris that may block water flow in drain or roof gutters				
F. 6	Proper disposal of items that can collect rainwater (i.e. bucket, discarded item such as cans, cups, bottles, food container, linoleum, old tyres etc.)				
F. 7	Proper disposal of household garbage				
F. 8	Clean up surrounding house area				
F. 9	Take mosquito preventive measures before going on long holidays such as covering all water containers, emptying water containers etc.				
**Prevention of mosquito bites**					
F.10	Sleep in mosquito net or have mosquito screens on windows				
F.11	Use mosquito coil, electric mosquito mat, liquid vaporizer, mosquito bulb, or mosquito trap				
F.12	Spraying dark places with an insecticidal spray				
F.13	Use mosquito repellent on body				
F.14	Avoid dark areas in the home where there is no light and no wind				
F.15	Wear long-sleeved shirts and pants to avoid mosquito bites				
F.16	Wear long-sleeved shirts and pants to avoid mosquito bites				
**Prevention of dengue transmission**					
F.17	Take measures to prevent mosquitoes from biting a dengue patient				
F.18	Put a dengue patient under bed nets				
F.19	Avoid sexual intercourse with spouse IF he/she infected with dengue fever.				

KNOWLEDGE, ATTITUDE AND PRACTICES REGARDING DENGUE FEVER AMONG ORANG ASLI COMMUNITY IN MALAYSIA (POST-INTERVENTION)

**Section D: Health Belief Model**					
D. 1	Do you think dengue is dangerous? Please state from scale 1(not dangerous) to 10 (very dangerous)	0 1 2 3 4 5 6 7 8 9 10<br>NOT dangerous ... VERY dangerous			
D. 2	From scale 1 (very unlikely) to 10 (highly likely), how do you rate your likelihood of getting dengue (risk of getting dengue)?	0 1 2 3 4 5 6 7 8 9 10<br>VERY unlikely ... HIGH likely			
D. 3	From scale 1 (no barriers) to 10 (high barrier), how do you rate the barriers you face to prevent dengue?	0 1 2 3 4 5 6 7 8 9 10<br>NO barriers at all ... HIGH barrier			
D. 4	From scale (Definitiely do no take action) to 10 (Definitely do the action), how do you rate any cues to action – incidence that motivate someone to carry out the prevention practices like death of family member due to dengue, encouragement from NGO, neighborhood infection with dengue.	0 1 2 3 4 5 6 7 8 9 10<br>DO NOT take action ... DO the action			
D. 5	From scale 1 (very not confident) to 10 (very confident), please rate your confidence level to carry out dengue prevention activities.	0 1 2 3 4 5 6 7 8 9 10<br>No confident at all ... VERY confident			
**Section E: Knowledge Related to Dengue**					
	**Statements**		**Yes**	**No**	**Don't Know**
**General Knowledge about dengue and *Aedes* Mosquito**					
E. 1	Dengue is transmitted by mosquito				

E. 2	The dengue virus is transmitted by *Aedes* Mosquito			
E. 3	Dengue Fever may become Dengue Hemorrhagic Fever			
E. 4	Dengue hemorrhagic fever can be fatal			
E. 5	Dengue Hemorrhagic Fever usually occurs in people who have had several dengue infections			
E. 6	Aedes mosquitos have black and white stripes on its leg and body			
E. 7	Aedes mosquito breeds in clean and stagnant water			
E. 8	Aedes mosquito prefers to live in the house or building rather than in natural wetlands			
E. 9	Aedes mosquito prefers to live in places with a lot of plants			
E.10	Aedes mosquitoes mainly bite during dusk and dawn			
**Knowledge about the transmission of dengue**				
E.11	The Aedes mosquitoes biting an infected person can spread it to another person			
E.12	Dengue fever usually appear 4 to 7 days after someone has been bitten by mosquito			
E.13	Dengue disease can be transmitted from an infected person by<br>touching			
E.14	Dengue disease can be transmitted from an infected person by Air			
E.15	Dengue disease can be transmitted from an infected person by Body fluid (saliva, sweat, semen)			
E.16	Dengue disease can be transmitted from an infected person by Blood			
E.17	Aedes mosquitoes' eggs can contain the dengue virus			
E.18	A person who has had dengue fever cannot get the infection again			
E.19	Dengue epidemic occurs only during the rainy season			
**Knowledge about prevention**				
E.20	Breeding of Aedes mosquitoes in the house can be prevented by<br>weekly change of stagnant water (pet bowls, flower pots or vases,etc.) in and around the house.			
E.21	Breeding of Aedes mosquitoes in the house can be prevented by<br>adding Abate/chemical in water containers			
E.22	Breeding of Aedes mosquitoes in the house can be prevented by covering water containers			

E.23	Breeding of Aedes mosquitoes in the house can be prevented by periodically emptying or drying out containers (old tires, trash cans, coconut shell etc) that retain water in and around the house			
E.24	Breeding of Aedes mosquitoes in the house can be prevented by proper disposal of items that can retain water (old tires, trash cans, coconut shell etc) around the house			
**Signs and symptoms of dengue fever**				
E.25	The high fever for 5 to 6 days			
E.26	Chills			
E.27	Rash			
E.28	Pain in the eyes			
E.29	Joint pain			
E.30	Headache			
E.31	Nausea and vomiting			
**Signs and symptoms of Dengue Hemorrhagic Fever (DHF)**				
E.32	Small red or purple spots under the skin			
E.33	Bleeding in the nose			
E.34	Bleeding in the gums			
E.35	Blood in stool			
E.36	Blood in urine			
E.37	Shortness of breath			
E.38	Dizziness or fainting			
**Knowledge about treatment, curability and precaution measures for people infected with dengue**				
E.39	There is no medication for treating dengue			
E.40	Immediate treatment can only prevent complications and death			

**Section F: Practices Regarding Dengue Prevention**					
	**Statements**	**Not at all**	**Rarely**	**Sometimes**	**Often**
**Prevention of Mosquito Breeding**					
F. 1	Cover all water containers used for storing water in or outside the house				
F. 2	Change stored water i.e water in water tank, pails, bathtub, flower vases, money plant, plates that support plant pots, refrigerator drip tray, plastic container on the bottom of kitchen cabinet/table legs (for controlling ants), etc.				

F. 3	Put Abate or chemical in water storage containers				
F. 4	Examine for mosquito larvae in containers for storing water, eg. pails, flower vases, water in money plant, plates that support plant pots, refrigerator drip tray, plastic container on the bottom of kitchen cabinet/table legs, etc.				
F. 5	Clear out debris that may block water flow in drain or roof gutters				
F. 6	Proper disposal of items that can collect rainwater (i.e. bucket, discarded item such as cans, cups, bottles, food container, linoleum, old tyres etc.)				
F. 7	Proper disposal of household garbage				
F. 8	Clean up surrounding house area				
F. 9	Take mosquito preventive measures before going on long holidays such as covering all water containers, emptying water containers etc.				
**Prevention of mosquito bites**					
F.10	Sleep in mosquito net or have mosquito screens on windows				
F.11	Use mosquito coil, electric mosquito mat, liquid vaporizer, mosquito bulb, or mosquito trap				
F.12	Spraying dark places with an insecticidal spray				
F.13	Use mosquito repellent on body				
F.14	Avoid dark areas in the home where there is no light and no wind				
F.15	Wear long-sleeved shirts and pants to avoid mosquito bites				
F.16	Wear long-sleeved shirts and pants to avoid mosquito bites				
**Prevention of dengue transmission**					
F.17	Take measures to prevent mosquitoes from biting a dengue patient				
F.18	Put a dengue patient under bed nets				
F.19	Avoid sexual intercourse with spouse IF he/she infected with dengue fever.				
